# Premenstrual Syndrome among Medical Students of a Medical college: A Descriptive Cross-sectional Study

**DOI:** 10.31729/jnma.8136

**Published:** 2023-04-30

**Authors:** Sunita Bhandari, Yam Dwa, Meenu Maharjan, Smrity Maskey, Minaxi Thakur, Santosh Sharma

**Affiliations:** 1 Department of Obstetrics and Gynecology, KIST Medical College and Teaching Hospital, Mahalaxmi, Lalitpur, Nepal

**Keywords:** *premenstrual syndrome*, *prevalence*, *quality of life*

## Abstract

**Introduction:**

Premenstrual syndrome is the premenstrual disorder with wide range of prevalence world-wide leading to higher rates of work absences, higher medical expenses, and lower health-related quality of life. The aim of this study was to find out the prevalence of premenstrual syndrome among medical students of a medical college.

**Methods:**

A descriptive cross sectional study was conducted in a medical college among medical students using self-reported questionnaires based on American College of Obstetricians and Gynaecologists criteria for premenstrual syndrome, and 12-Item Short Form Health Survey for quality of life, from 1 January 2022 to 31 March 2022 after obtaining ethical approval from the Institutional Review Committee (Reference number: 207807955). Convenience sampling was used among students who met the inclusion criteria. Point estimate and 95% Confidence Interval were calculated.

**Results:**

Among 113 patients, premenstrual syndrome according was seen in 83 (73.45%) (82.9383.06, 95% Confidence Interval) out of which, 56 (67.46%) showed mild premenstrual syndrome, and 27 (32.53%) had moderate premenstrual syndrome. The most commonly reported affective symptoms of premenstrual syndrome was irritability 82 (98.79%), while the somatic symptoms was abdominal bloating 63 (75.90%).

**Conclusions:**

The prevalence of premenstrual syndrome among medical students was similar to in the other studies done in similar settings.

## INTRODUCTION

Premenstrual Syndrome (PMS) is the premenstrual disorder with a wide range of prevalence world-wide.^1^ A Systematic Review and Meta-analysis based on different parts of the world records the pooled prevalence of PMS to be 47.8% with a range between 12% to 98%.^2^

The burden of disease is high, women with PMS have higher rates of work absences, higher medical expenses, and lower health-related quality of life.^3^ Premenstrual disorders are also common among young girls leading to poor health-related quality of life.^4,5^

The aim of this study was to find out the prevalence of premenstrual syndrome among medical students of a medical college.

## METHODS

This descriptive cross sectional study was carried out among female undergraduate medical students, after taking written consent at KIST Medical College and Teaching Hospital, enrolled in the MBBS program from 1 January 2022 to 31 March 2022 after obtaining ethical approval from the Institutional Review Committee (Reference number: 207807955). The medical students having a regular menstrual period at least in the last six consecutive months, enrolled full-time in undergraduate studies, unmarried were included in the study. While medical students having irregular menstrual cycle, currently pregnant, history of chronic illness, diabetes, high blood pressure, heart disease, thyroid disorder, history of current depression, anxiety, and any other psychiatric disorders, currently using a hormonal method of contraception were excluded from the study. Convenience sampling method was used. Total female students during the study period were 159 (54 in final year, 41 in fourth year, 33 in third year, and 31 in second year). The sample size was calculated using following formula:


$$\begin{array}{ll} \text{n} & ={\text{Z}}^{2}\times \frac{\text{p}\times \text{q}}{{\text{e}}^{\text{2}}}\\ & =1.96^{2}\times \frac{0.50\times 0.50}{0.05^{2}}\\ & =385 \end{array}$$

Where,

n = minimum required sample size
Z = 1.96 at 95% Confidence Interval (CI)
p = prevalence taken as 50% for maximum sample size calculation
q = 1-p
e = margin of error, 5% n

$$\begin{array}{ll} \text{n'} & =\frac{\text{n}}{1+\frac{\text{(n}-1)}{\text{N}}}\\ & =\frac{\text{385}}{1+\frac{\text{385}-1}{\text{159}}}\\ & =113 \end{array}$$

Where,

n'= adjusted sample size for a finnite population
N = Total study population

Thus the final adjusted sample sie was 113. Selfreported questionnaires was used which include the basic demographic and menstrual information of the participants. It consists of questionnaire incorporating all the PMS symptoms. The diagnosis of PMS was based on criteria listed by American College of Obstetrics and Gynecology (ACOG).^1^ PMS was diagnosed if at least one of the 6 affective (depression, angry outbursts, irritability, anxiety, confusion, social withdrawal) and one of these somatic symptoms (abdominal bloating, breast tenderness, headache, Joint or muscle pain, swelling of extremities, weight gain) reported five days prior to the onset of menses in the three prior menstrual cycles and ceased within four days of onset of menses. The response to each PMS symptoms was scored as 0 for 'not at all' and 3 for 'severe' and the highest indicated score was used for calculation. A total score was calculated by summing the symptom scores and dividing by the mean number of symptoms, converting it to a percentage. Mild, moderate and severe PMS corresponded to 0-33%, 33-66% and >66% respectively. It also includes the checklist to mark whether the symptoms occurred one week before periods or after periods or due to other reasons The questionnaires adopted from Shorter Form (SF-12) health survey^6^ which has been widely used in many research fields to measure general quality of life was also used. It is a brief, well-validated and reliable generic questionnaire to measure general health status, and outcome information. It contains 12 items that measures physical and mental components of health (psychological distress and psychological well-being). Physical component consists four sub groups: physical functioning, role limitation due to physical problems, bodily pain, general health perceptions; and Psychological component consists four sub groups: social function, general mental health, role limitation due to emotional problems and vitality. It also provides psychometrically-based physical component summary (PCS) and mental component summary (MCS) scores. The SF-12 is scored so that a high score indicates better quality of life.

Data were analyzed using IBM SPSS Statistics version 25.0. Point estimate and 95% CI were calculated.

## RESULTS

Among 113 female students, the prevalence of PMS was 83 (73.45%) (82.93-83.06, 95% CI). Out of which, 56 (67.46%) showed mild PMS (Table 1).

**Table 1:** PMS according to ACOG criteria (n= 83).

Category	n (%)
Mild PMS	56 (67.46)
Moderate PMS	27 (32.53)
Severe PMS	-

Severe PMS-The mean age of the female participants was 22.7±1.3 years (Table 2).

**Table 2:** Demographic and menstrual characteristics of study population with PMS (n= 83).

Variables	mean±SD
Age (years)	22.7±1.30
BMI (kg/m2)	21.9±2.90
Menarche (years)	12.9±1.30
Length of menstrual cycle (days)	30.5±4.80
Duration of flow (days)	4.6±1.20

Dysmenorrhea was present among 70 (84.3%) students. A total of 20 (24.1%) student had self-medication and 4 (4.80%) were absent from the college and 1 (1.20%) seek the doctor consultation (Table 3).

**Table 3:** Intensity of dysmenorrhea (n= 70).

Intensity of dysmenorrhea	n (%)
Mild	65 (51.18)
Moderate	38 (45.8)
Severe	13 (15.7)

The most commonly reported affective symptoms with PMS were Irritability 82 (98.79%), Angry outburst 73 (87.95%), Anxiety 57 (68.67%) while the most commonly reported somatic symptoms were abdominal bloating 63 (75.90), breast tenderness 58 (69.87), Joint or muscle pain 47 (56.62%) (Table 4).

**Table 4:** Premenstrual symptoms in PMS (n = 83).

Symptoms	n (%)
**Affective Symptoms**	
Depression	9 (7.08)
Angry outburst	73 (87.95)
Irritability	82 (98.79)
Anxiety	57 (68.67)
Confusion	36 (43.37)
Social Withdrawal	50 (60.24)
**Somatic Symptoms**	
Breast tenderness	58 (69.87)
Abdominal bloating	63 (75.90)
Headache	42 (50.60)
Swelling of extremities	9 (10.84)
Joint or muscle pain	47 (56.62)
Weight gain	19 (22.89)

The mean value of PCS among student with PMS was 47±6.8 (Table 5).

**Table 5:** SF-12 outcome measures in study population with PMS (n = 83).

Variables	mean±SD
PCS	47±6.80
MCS	43.30±9.70

## DISCUSSION

The prevalence of PMS according to ACOG criteria in our study was 83 (73.45%), among which 56 (67.46%) showed mild PMS, 27 (32.53%) showed moderate PMS and none of them had severe form. This findings is consistent with the previous study done in Nepal (2017),^7^ which reported prevalence of PMS to be 61.1%. This is also consistent with study done in KSA (2014),^4^ reported the prevalence of PMS as 78.5%. This study is not in agreement with the study done in India (2014),^8^ where only 22.3% were reported to have PMS. Also differs from the study done in Japan (2017),^9^ showed low prevalence of PMS as 25.2%. Another study in Nepal (2019),^10^ also showed 17.2% moderate to severe PMS with the rest 80.7% having no or mild PMS. The differences in prevalence could be due to the difference in the used questionnaire to assess PMS. Variation could also be due to differences in sample size as well as differences in biological, social, cultural and geographical factors among studies.

In our study, the frequency of PMS was found to be higher in students with history of dysmenorrhea 70 (84.3%) which is similar to previous studies.^4,8,10-12^

The most commonly reported affective symptoms with PMS were Irritability 82 (98.79%), angry outburst 73 (87.95%) while the most commonly reported somatic symptoms were abdominal bloating 63 (75.90), breast tenderness 58 (69.87) which is similar to previous studies done in india^8,13^ and Turkey.^12^ In a study done in medical students in India, the most frequent affective symptom was irritability (35%) and somatic symptom was breast tenderness (41%).^14^ The commonest affective symptom was irritability (81.2%) and somatic symptom was headache (77%) in a study conducted in another area of Nepal.^7^

The mean PCS and MCS score among student with PMS was 47±6.80 and 43.30±9.70 which is lower. Similarly, study done in Pakistan (2008) among medical students showed PCS and MCS scores were significantly lower in the PMS group.^15^ Study among medical students done in KSA (2014) showed scores in all subscales of SF-36 in the PMS group was significantly lower than those without PMS except for physical functioning subscale.^4^ Previous studies done in Turkey (2014),^11^ (2015),^12^ India (2019)^16^ and Brazil (2019)^5^also found significantly lower quality of life among students with PMS. This study showed that the burden of PMS on health-related quality of life was both on mental and physical health-related quality of life.

Our findings have some limitations because the population of this study included a highly selective sample of medical students from one academic institute, which will limit the generalization of the findings. Also questionnaire filling is likely to pose some bias during recall of symptoms.

## CONCLUSIONS

The prevalence of premenstrual syndrome among medical students was similar to other studies done in similar settings. Quality of life among students with PMS was poor thus strategies should be adopted for timely detection and effective management of PMS to ensure their well-being and efficiency.
